# Interventions to promote patients and families’ involvement in adult intensive care settings: a protocol for a mixed-method systematic review

**DOI:** 10.1186/s13643-019-1102-9

**Published:** 2019-07-25

**Authors:** Andreas Xyrichis, Simon Fletcher, Sally Brearley, Julia Philippou, Ed Purssell, Marius Terblanche, Anne Marie Rafferty, Scott Reeves

**Affiliations:** 1 Florence Nightingale Faculty of Nursing, Midwifery and Palliative Care, Kings College London, James Clerk Maxwell Building, Waterloo, London, UK; 2 Kingston and St. George’s University London, St. George’s University, Hunter Wing 6th Floor, Tooting, London, UK; 3 Guy’s and St. Thomas’ NHS Foundation Trust, Lambeth, London, UK

**Keywords:** Systematic review, Intensive care, Family member involvement, Typology

## Abstract

**Background:**

There has been an identified need for greater patient and family member involvement in healthcare. This is particularly relevant in an intensive care unit (ICU), as the family provides a key communicative and practical link between patient and clinician. Family members have been deemed a positive beneficial influence on ICU care and recovery processes, yet they themselves are often emotionally affected after discharge. There has been no standardised evidenced-based approach which explores research on family member involvement and the range and quality of contributions remain unclear. This project will undertake a systematic review to assess the evidence base for interventions designed to promote patient and family member involvement in adult intensive care settings and develop a comprehensive typology of interventions for use by clinicians, patients and carers.

**Methods:**

The following databases will be searched without date restriction: MEDLINE, EMBASE and CINAHL, as well as the Cochrane Central Register of Controlled Trials, Joanna Briggs and Cochrane Libraries. Manual searches of recent back issues of leading ICU and patient experience journals will also be undertaken, as will the reference lists of included studies. Unpublished literature will be sought through grey literature databases, including GreyLit and OpenGrey. All evaluation studies that consider intervention activities to promote patient and family member involvement in adult ICUs will be included; all research designs will be eligible. We will seek to include studies that report on a mixture of relevant outcomes for patients and family members. Abstracts and papers will be independently screened by at least two members of the team to determine their inclusion. Included papers will be assessed for methodological rigour using a standard rating approach, which assesses ‘quality of study’ and ‘quality of information’. Quality assessment will be completed by at least two members of the team. Data on interventions, evaluation methods and outcomes will be collated using a predetermined extraction table. These are likely to be heterogeneous in nature, which will mean that the review will follow a narrative approach to synthesis.

**Discussion:**

The review will provide valuable and rigorous insight into the range and quality of interventions available to promote patient and family member involvement in ICU. This is the first step towards addressing the absence of a synthesis of research for this context, and will, in addition, develop a typology of available interventions that will help service users and clinicians make informed decisions about the approaches to patient and family member involvement which they might want to adopt.

**Systematic review registration:**

PROSPERO (CRD42018086325).

**Electronic supplementary material:**

The online version of this article (10.1186/s13643-019-1102-9) contains supplementary material, which is available to authorized users.

## Background

There have been increasing calls from policymakers and the public over a number of years for greater patient and family member involvement in healthcare [1–3]. The current drive can be traced back to a seminal Institute of Medicine [1] report in the USA that made a strong call for greater patient-centred care, which included keeping patients and families informed and actively involved in medical decision-making and care management in order to reduce the risk of medical error and drive up quality. Similarly, in the UK, Wanless [2] put forward the notion of a ‘fully-engaged’ public involved in their care. However, a decade later it was noted that this aspiration had still not been fully realised in the National Health Service (NHS) and that there was a continued paternalism in care delivery with only limited opportunities for user involvement [4].

Despite the changing economic climate since Wanless [2], the principles of self-care, participation and collaboration between patients, their families and professionals have continued to be promoted by professionals and policy makers [3, 5, 6]. Indeed, following the fall-out from the Francis Report [5] in the UK and other commissioning directives [6], the NHS is re-organising itself with patients being seen as a central part of delivering collaborative care to ensure quality and efficiency. The concern with patient and family member involvement persists and was echoed in the recent NHS 5-Year Forward View [3] report, which endorsed the view that many people wish to be more informed about, and involved with, their own care. For the purposes of this systematic review protocol, patient and family member involvement refers to the interaction of different professionals to improve care by ensuring patients and their families are involved in decision-making, sharing of information, power and responsibility for patient needs and choices.

The notion of patient and family member involvement is especially relevant in the intensive care unit (ICU), where it can have profound consequences for care decisions, delivery and outcomes; this has recently been identified as a priority area for ICU in the UK by the James Lind Alliance through a priority setting partnership involving patients, carers and clinicians [7]. This is partly because ICU patients are rarely in a position to communicate directly with clinicians and recollect their ICU experience, which means that the responsibility for this often lay with their family members. While this is a UK-wide concern, a recent mixed-methods study found great variation in family satisfaction across ICUs in England, Wales and Northern Ireland [8].

Studies over the past decade have confirmed that family members can not only have a positive influence on a patient’s care and recovery from ICU but also that family members themselves can be affected even after discharge [9–14]. In particular, within the first few days after ICU admission, family members can show signs of anxiety, depression and stress; report difficulties in understanding the information clinicians try to communicate with them; and those who suffered a bereavement are at risk of generalized anxiety, panic attacks, depression and post-traumatic stress syndrome (PTSD) [10]. For example, Azoulay et al. [9] surveyed family members of patients who have been in ICU to find that 90 days after discharge more than a third (34%) of them suffered from PTSD symptoms. In addition, they noted higher rates (48%) among those family members who indicated the information they were given was incomplete. Furthermore, adequate patient and family member involvement could also have implications for the longer-term rehabilitation of ICU patients. Bench et al. [11] found through focus groups with general practice (GP) staff and former ICU patients in England that the responsibility for sharing information about an ICU stay is often left to the patient, and in many cases to a family member. In that study, family members were seen to have a key role to play in facilitating a successful transition from ICU to home yet were rarely adequately involved or prepared for taking on this role. Challenges around patient and family member involvement can also affect ICU staff, who for example have reported increased stress and anxiety when communication is poor between families and clinicians [12]. Finally, patient and family member involvement is not limited to issues of communication or decision-making; studies have shown families to be keen to, and clinicians to be happy for them to, support with essential care activities like bathing, mouth care, eye care, repositioning and feeding [14].

While there is a growing research body on patients, family members and clinicians’ perceptions of involvement in ICU decision-making and care activity, we are still missing a standardised, evidence-based, internationally relevant approach to facilitate this process. Indeed, a recent scoping review by Olding et al. [13] sought to investigate the extent and range of literature on this topic, finding evidence of growth in papers with over 100 reports of different qualitative and quantitative designs identified. They identified interventions which aimed to integrate family members into rounds processes, enhance patient involvement, evaluate environmental factors with a view towards patient-centred care, develop communication opportunities and contribute to care using physical intervention. Examples of interventions include family member participation in medical rounds, communication aids and physical contributions to care such as massage. However, they did not seek to rate the quality of the evidence nor did they examine the type of interventions available, and conditions under which these can be effective. While this is an area of growing interest, we are still unclear about the range and quality of interventions available to promote patient and family member involvement in ICU. The current systematic review protocol is the first step towards addressing this global issue, the results of which will help service users make informed decisions about approaches to involvement they might want to adopt, depending on their circumstances. Given the diverse nature of research designs and evidence available on this topic, a mixed-method systematic review is outlined below.

## Objectives

We will complete a systematic review of interventions designed to promote patient and family member involvement in adult intensive care settings and, in doing so, we will develop a classification framework/matrix, known as a typology, of existing interventions that take into account their effectiveness and quality of their evidence base. This typology can be utilised by ICU clinicians to inform their decision-making for adopting a particular approach to involvement for their units; and considered by ICU patients and their relatives who may wish to be more involved in their care. Our systematic review will answer the following question: What are the available interventions, and which are most effective, for including patients and family members in the care processes and decisions made in adult intensive care settings? Our objectives include (1) undertaking a comprehensive and systematic search of published and unpublished studies reporting on interventions that promote involvement in intensive care; (2) robustly assess the quality of empirical evidence for all included studies; (3) generate a detailed description and synthesis of interventions and their associated outcomes; (4) classify interventions and outcomes in order to develop a typology of interventions, outlining key factors that support or impede involvement; and (5) develop a logic model, that draws from the literature to hypothesise the mechanisms through, and conditions under which such interventions can be effective.

## Methods

The current systematic review protocol follows the Preferred Reporting Items for Systematic Reviews and Meta-Analyses Protocol (PRISMA-P) guidelines [15] (Additional file 1). To ensure transparency of the methods employed, our review protocol was registered at the International Prospective Register of Systematic Reviews (PROSPERO) database (CRD42018086325).

We have built five distinct components to this review: (i) study eligibility criteria; (ii) search for evidence; (iii) data management, screening and extraction; (iv) quality assessment; (v) data analysis and synthesis.

### Study eligibility criteria

#### Study designs

Given the diverse nature of available evidence, we will include evaluation studies of any design. With regard to identification of available interventions, designs may include case studies, action research and ethnographic approaches. With regard effectiveness of interventions, designs may include randomised controlled trials, quasi-experimental and cohort studies.

#### Setting and participants

Any study undertaken with clinicians, patients or carers based in adult intensive care will be considered. Paediatric settings will not be part of this review due to the unique features that characterise child/parent relationships, where participation and involvement can take on different meanings.

#### Interventions

In the context of this review, interventions are likely to be multifaceted and complex. We will consider reports of any kind of activities as long as they are intended to promote the participation of patients/carers/family members in adult intensive care. Studies that might report a component relating to involvement as part of a larger intervention will also be considered, where that component can be discerned.

#### Comparisons

Given the broad perspective taken on the interventions to be considered in this review, we will not exclude studies based on comparisons, which might vary or reported as standard practice. Any limitations related to poor reporting of comparisons will be reflected accordingly in the quality assessment of papers.

#### Outcomes

We will seek to include studies that report on a mixture of relevant outcomes for patients and family members, which may include standard measures such as quality of life (e.g. SF36), Impact of Event Scale, Hospital Anxiety and Depression Scale and any non-standard but patient-sensitive indicators such as patient/relative satisfaction. Where outcomes are reported as a composite measure, we will seek to extract all composite and individual outcomes of relevance. Outcomes will be extracted as reported, in all data forms including continuous, dichotomous and narrative formats.

#### Timing and language

To maximise coverage of the existing literature worldwide, we will seek to include relevant studies of any duration and publication date. Articles published in any language will be included.

### Search for evidence

We will search an extensive range of sources to ensure comprehensive coverage of the literature. We will perform database searches in MEDLINE (OVID interface, 1948 onwards), EMBASE (Ovid interface, 1980 onwards), CINAHL (EBSCO interface, 1982 onwards) and the Cochrane Central Register of Controlled Trials (Wiley interface, latest issue) using a combination of medical subject headings (MeSH) and free-text keywords related to family member involvement in ICU (Additional file 2). The Cochrane Library and the Joanna Briggs Library will also be searched for existing reviews.

The database searches will be complemented by searches for clinical studies through the UK Clinical Trials Gateway (www.ukctg.nihr.ac.uk), US NIH clinical studies registry (clinicaltrials.gov), EU Clinical Trials Register (www.clinicaltrialsregister.eu), WHO International Clinical Trials Registry Platform (apps.who.int/trialsearch) and the International Standard Randomised Controlled Trial Number–ISRCTN registry (www.isrctn.com).

To ensure wide coverage, we will also hand search recent back issues of key critical care e-journals (e.g. Critical Care, Journal of Critical Care, Intensive and Critical Care Nursing), scan reference lists of identified reviews and included articles; search our personal libraries and consult with experts in the field. GoogleScholar will also be searched. Non-commercially published (Grey) literature will also be sought through OpenGrey (www.opengrey.eu) and the GreyLit Report (www.greylit.org) databases.

### Data management, screening and extraction

Search results will be imported into Covidence (www.covidence.org), the standard production platform for Cochrane Reviews. Covidence is an online software that facilitates collaboration among reviewers throughout the stages of the review including the screening process, data extraction and quality appraisal of studies. Based on our inclusion criteria, we will develop screening questions with relevant forms to import alongside the citations retrieved into Covidence. We will pilot these questions/forms before the formal screening process to check for reviewer consistency using a sample of 10 citations.

The citations retrieved from the searches will be screened by two reviewers who will independently screen papers in duplicate against the inclusion criteria. Papers that meet our criteria following title and abstract review will be independently read in full by two authors in duplicate to decide on their eligibility; if necessary, additional information will be sought from study authors. Any disagreement will be resolved through discussion or by involving a third reviewer. Reasons for excluding papers read in full will be recorded.

Standardised data extraction forms will be used by at least two reviewers to independently extract key information (Additional file 3). These will include the nature of the PFMI intervention, e.g. aims, objectives, activities, duration; reported outcomes, e.g. SF 36, Impact of Event Scale, Hospital Anxiety and Depression Scale, patient/carer satisfaction; and methods of evaluation, e.g. research designs, data collection methods, approaches to analysis, sampling. Any disagreements will be resolved through a consensus approach or the involvement of a third reviewer. We will contact study authors for any missing data or inadequately reported interventions. We will pilot these forms before the formal screening process to check for reviewer consistency using a sample of five papers.

### Quality assessment

Each included paper will be assessed for methodological rigour using an established rating approach utilised successfully in a number of previous mixed-method systematic reviews [16–18]. This rating system assesses ‘quality of study’ to consider: appropriate fit between study design/research questions, attention to ethical issues and use of appropriate analyses; and, ‘quality of information’ to assess for a clear rationale for the intervention, good contextual information and identification of possible biases (Additional files 4 and 5). Quality assessment will be undertaken independently by at least two reviewers. For each domain, we will describe the procedures reported in the paper, using verbatim quotes as necessary. A calibration exercise using a sample of five papers will be undertaken to check for reviewer consistency.

### Analysis and synthesis

We will summarise search results using the PRISMA flow diagram. The overall approach to the analysis and synthesis will follow a segregated methodologies approach, which is becoming a standard method adopted in mixed-method reviews [19]. Accordingly, a separate synthesis of quantitative and qualitative data will be initially completed followed by a mixed-method synthesis, as described below.

Quantitative studies will be grouped depending on study design, and results will be summarised using descriptive statistics including frequencies, percentages, means and medians depending on type of data and distribution; where appropriate, a meta-analysis will be explored. Attention will be paid to the methodological and clinical heterogeneity among the studies, and a random or fixed effects model will be applied as needed. Where heterogeneity precludes statistical combination through a meta-analysis, a narrative approach to synthesis will be pursued.

Synthesis of qualitative data will follow the best-fit framework approach [20]. First, we will identify a theoretical model on PFMI to guide data coding and synthesis. In the event that the team cannot agree or identify a suitably adequate theoretical model, we will develop one. We will also seek to develop clearer definitions for family as a unit and family member as a subgroup of a unit. Second, papers will be read and coded using the model and its core concepts as provisional categories. All sections of the papers will be reviewed for coding. Themes not accounted for by the model will be noted, coded and classified under a new category. Third, any additional themes/categories uncovered will be considered by the team and used to supplement the original model. If the model changes substantially from the original, the papers will be re-coded based on the new model. We will use the model to inform but not restrict data synthesis, with additional themes not captured by the model used to challenge and add to previously held assumptions.

The mixed-method synthesis will utilise the quantitative data to inform on the measured effects, while the qualitative data will inform on the perceived effects. In this way, the qualitative and quantitative data will act as complementary rather than as confirmatory of each other. A key element of the mixed-method synthesis will be to classify interventions and outcomes in order to develop a typology of family involvement in ICU that will present the key factors that support or impede involvement. Given the aim of the typology is to classify dimensions and types that identify and describe the phenomena under analysis, we will produce a ‘descriptive typology’ [21]. Specifically, the typology will provide a clear categorisation of the different interventions used in the ICU and their related activities, materials, participants, contexts and evidence of effectiveness. We will employ well-established social science techniques to develop the typology, which involve the following tasks: forming and refining key issues, drawing out underlying dimensions, creating categories for classification and sorting cases [22]. The typology will be illustrated in a matrix-like format and include interventions supported by either suggestive or firm evidence, but clearly distinguish between these. With suggestive evidence, we will refer to evidence from non-randomised experiments, pre/post-test, cohort studies and case series. Evidence from a minimum of one high-quality randomised controlled trial, with low risk of bias, will be regarded as firm evidence.

## Discussion

By pursuing the question: what are the available interventions, and which are most effective, for including patients and family members in the care processes and decisions made in adult intensive care settings? This review will explore the range and quality of interventions available to promote patient and family member involvement in ICU by adopting an approach which is both valuable and rigorous. The systematic review represents the first step towards addressing the previous absence of a synthesis of research for this context, and will, in addition, help service users make informed decisions about their potential adoption of approaches to patient and family member involvement in real terms.

Specifically, this systematic review will synthesise and appraise evidence on existing patient and family member involvement activities and will lead to (1) clarity about the best evidence available of how to effectively include patients and family members in their care; (2) a richly informed insight into factors that impede or foster involvement in intensive care settings; (3) knowledge about the organisational conditions and context that can enable different kinds of involvement activities to be successful; (4) identification of common study pitfalls and weaknesses related to involvement in ICU; and (5) development of a robust, empirically based and comprehensive typology of interventions, which can be used as a guide to action for clinicians and patients/family members, and can inform decisions about enabling greater patient involvement in ICU care.

Results from this work will generate a comprehensive picture of what involvement activities are available from the international literature, which have been shown to work so far and under what conditions. This will enable us to develop a typology of interventions that promote family member involvement in ICU that takes into account their effectiveness, quality of their evidence base, as well as factors that support or impede involvement and conditions under which activities are more likely to be successful. Even though our results will draw from the literature, they will still serve as a very helpful tool to support patients who survive ICU and their family members to make more informed decisions about their involvement to their care.

It is anticipated that we will use the learning gained from the systematic review to inform the development of a follow-up study to empirically examine and contextualise this typology in different ICU settings. We expect that this follow-up study will ensure our systematic review results have been robustly ‘road tested’ within a range of ICU settings. This in turn will produce a rigorous and useable tool/intervention that can help service users make informed decisions about approaches to involvement they might want to adopt, depending on their circumstances and preferences. This tool/intervention can enable the provision of better and more efficient intensive care services, ultimately improving both short- and longer-term outcomes for patients who survive ICU and their family members.

Through this systematic review of the literature, we can ensure ICU clinicians, patients, family members and researchers’ efforts are not wasted or misplaced; but are directed at interventions that have a stronger evidence base and greater potential for success. The insight to be gained through this review will place us and other research teams in a better place to make informed decisions about the design of future research work and development of evidence-based guidelines for promoting patient and family member involvement in ICU.

## Additional files

Additional file 1: PRISMA-P checklist. (DOC 85 kb)

Additional file 2: Draft—database search strategy. (DOCX 13 kb)

Additional file 3: Draft—data extraction form. (DOCX 17 kb)

Additional file 4: Draft—quantitative quality assessment form. (DOCX 19 kb)

Additional file 5: Draft—qualitative quality assessment form. (DOCX 16 kb)

## Data Availability

The datasets generated and/or analysed during the current study are available from the corresponding author on reasonable request.
