# Supplementary material for: Interventions to promote patients and families’ involvement in adult intensive care settings: a protocol for a mixed-method systematic review
Source: Syst Rev. 2019 Jul 25;8:185. doi: 10.1186/s13643-019-1102-9 (PMC6657078; doi:10.1186/s13643-019-1102-9)
Supplement: Supplementary file 1 — PRISMA-P checklist. (DOC 85 kb) [file 13643_2019_1102_MOESM1_ESM.doc]

**PRISMA-P (Preferred Reporting Items for Systematic review and Meta-Analysis Protocols) 2015 checklist: recommended items to address in a systematic review protocol***

| Section and topic | Item No | Checklist item |  |
| --- | --- | --- | --- |
| ADMINISTRATIVE INFORMATION | | |  |
| Title: |  |  |  |
| Identification | 1a | Identify the report as a protocol of a systematic review | The effectiveness of interventions to promote patients' and families' involvement in adult intensive care settings: a systematic review. |
| Update | 1b | If the protocol is for an update of a previous systematic review, identify as such | Not applicable |
| Registration | 2 | If registered, provide the name of the registry (such as PROSPERO) and registration number | In accordance with the guidelines, our systematic review protocol was registered with the International Prospective Register of Systematic Reviews (PROSPERO) on 20/2/18 and was last updated on 20/12/18 (registration number CRD42018086325) |
| Authors: |  |  |  |
| Contact | 3a | Provide name, institutional affiliation, e-mail address of all protocol authors; provide physical mailing address of corresponding author | **Andreas Xyrichis (corresponding author)**: Kings College London, James Clerk Maxwell Building, Waterloo, London, [andreas.xyrichis@kcl.ac.uk](mailto:andreas.xyrichis@kcl.ac.uk)  **Scott Reeves**: Kingston & St Georges University London, Hunter Wing 6^th^ Floor, St Georges University, Tooting, London, [s.reeves@sgul.kingston.ac.uk](../s.reeves@sgul.kingston.ac.uk)  **Simon Fletcher**: Kingston & St Georges University London, Hunter Wing 6^th^ Floor, St Georges University, Tooting, London, [simon.fletcher@sgul.kingston.ac.uk](mailto:simon.fletcher@sgul.kingston.ac.uk)  **Sally Brearly**: Kingston & St Georges University London, Hunter Wing 6^th^ Floor, St Georges University, Tooting, London, [sally.brearley@icloud.com](../sally.brearley@icloud.com)  **Julia Philippou**: Kings College London, James Clerk Maxwell Building, Waterloo, London, [julia.philippou@kcl.ac.uk](mailto:julia.philippou@kcl.ac.uk)  **Ed Purssell**: Kings College London, James Clerk Maxwell Building, Waterloo, London, [ed.purssell@kcl.ac.uk](../ed.purssell@kcl.ac.uk)  **Marius Terblanche**: Kings College London, James Clerk Maxwell Building, Waterloo, London, [marius.terblanche@kcl.ac.uk](mailto:marius.terblanche@kcl.ac.uk)  **Anne Marie Rafferty**: Kings College London, James Clerk Maxwell Building, Waterloo, London, [anne_marie.rafferty@kcl.ac.uk](mailto:anne_marie.rafferty@kcl.ac.uk) |
| Contributions | 3b | Describe contributions of protocol authors and identify the guarantor of the review | AX is the guarantor. AX, SR and SF drafted the manuscript. All authors contributed to the development of the selection criteria, the risk of bias assessment strategy and data extraction criteria. JP developed the search strategy. All authors read, provided feedback and approved the final manuscript |
| Amendments | 4 | If the protocol represents an amendment of a previously completed or published protocol, identify as such and list changes; otherwise, state plan for documenting important protocol amendments | If we need to amend this protocol, we will give the date of each amendment, describe the change and give the rationale in this section. Changes will not be incorporated into the protocol |
| Support: |  |  |  |
| Sources | 5a | Indicate sources of financial or other support for the review | This systematic review is funded by the National Institute for Health Research (funding reference number PB-PG-0416-20021; The effectiveness of interventions to promote patients' and families' involvement in adult intensive care settings: a systematic review. |
| Sponsor | 5b | Provide name for the review funder and/or sponsor | Guy's and St Thomas' NHS Foundation Trust |
| Role of sponsor or funder | 5c | Describe roles of funder(s), sponsor(s), and/or institution(s), if any, in developing the protocol | The National Institute for Health Research is funding the project. The NIHR is not involved in any aspect of the project. |
| INTRODUCTION | | |  |
| Rationale | 6 | Describe the rationale for the review in the context of what is already known | See Background (p.3) |
| Objectives | 7 | Provide an explicit statement of the question(s) the review will address with reference to participants, interventions, comparators, and outcomes (PICO) | See Background (p.4) |
| METHODS | | |  |
| Eligibility criteria | 8 | Specify the study characteristics (such as PICO, study design, setting, time frame) and report characteristics (such as years considered, language, publication status) to be used as criteria for eligibility for the review | See Methods (p.5) |
| Information sources | 9 | Describe all intended information sources (such as electronic databases, contact with study authors, trial registers or other grey literature sources) with planned dates of coverage | See Methods (p.6) |
| Search strategy | 10 | Present draft of search strategy to be used for at least one electronic database, including planned limits, such that it could be repeated | See Appendix 2 |
| Study records: |  |  |  |
| Data management | 11a | Describe the mechanism(s) that will be used to manage records and data throughout the review | See Methods (pp.6-7) |
| Selection process | 11b | State the process that will be used for selecting studies (such as two independent reviewers) through each phase of the review (that is, screening, eligibility and inclusion in meta-analysis) | See Methods (p XX) |
| Data collection process | 11c | Describe planned method of extracting data from reports (such as piloting forms, done independently, in duplicate), any processes for obtaining and confirming data from investigators | See Methods (p XX), Appendix 3 |
| Data items | 12 | List and define all variables for which data will be sought (such as PICO items, funding sources), any pre-planned data assumptions and simplifications | See Appendix 3 |
| Outcomes and prioritization | 13 | List and define all outcomes for which data will be sought, including prioritization of main and additional outcomes, with rationale | See Appendix 3 |
| Risk of bias in individual studies | 14 | Describe anticipated methods for assessing risk of bias of individual studies, including whether this will be done at the outcome or study level, or both; state how this information will be used in data synthesis | See Methods (p.7) |
| Data synthesis | 15a | Describe criteria under which study data will be quantitatively synthesised | Not applicable |
|  | 15b | If data are appropriate for quantitative synthesis, describe planned summary measures, methods of handling data and methods of combining data from studies, including any planned exploration of consistency (such as I^2^, Kendall’s τ) | Not applicable |
|  | 15c | Describe any proposed additional analyses (such as sensitivity or subgroup analyses, meta-regression) | See Methods (pp.7-8) |
|  | 15d | If quantitative synthesis is not appropriate, describe the type of summary planned | See Methods (pp. 7-8) |
| Meta-bias(es) | 16 | Specify any planned assessment of meta-bias(es) (such as publication bias across studies, selective reporting within studies) | See Methods (p.7) |
| Confidence in cumulative evidence | 17 | Describe how the strength of the body of evidence will be assessed (such as GRADE) | See Methods (p.7) |

*** It is strongly recommended that this checklist be read in conjunction with the PRISMA-P Explanation and Elaboration (cite when available) for important clarification on the items. Amendments to a review protocol should be tracked and dated. The copyright for PRISMA-P (including checklist) is held by the PRISMA-P Group and is distributed under a Creative Commons Attribution Licence 4.0.**

*From: Shamseer L, Moher D, Clarke M, Ghersi D, Liberati A, Petticrew M, Shekelle P, Stewart L, PRISMA-P Group. Preferred reporting items for systematic review and meta-analysis protocols (PRISMA-P) 2015: elaboration and explanation. BMJ. 2015 Jan 2;349(jan02 1):g7647.*
