# Supplementary material for: Interventions to promote patients and families’ involvement in adult intensive care settings: a protocol for a mixed-method systematic review
Source: Syst Rev. 2019 Jul 25;8:185. doi: 10.1186/s13643-019-1102-9 (PMC6657078; doi:10.1186/s13643-019-1102-9)
Supplement: Supplementary file 2 — Draft—database search strategy. (DOCX 13 kb) [file 13643_2019_1102_MOESM2_ESM.docx]

**Additional file 2: Draft – Database search strategy**

OvidSP – MEDLINE, 1948 onwards

1. exp Critical Care/

2. exp Intensive Care Units/

3. critical care.mp

4. intensive care.mp

5. or/1-4

6. exp Intensive Care, Neonatal/

7. exp Intensive Care Units, Pediatric/

8. exp Intensive Care Units, Neonatal/

9. pediatric*.mp

10. neonatal.mp

11. or/6-10

12. 5 not 11

13. exp Patients/

14. exp Family/

15. exp Caregivers/

16. patient*.mp

17. famil*.mp

18. carer*.mp

19. or/13-18

20. involve*.mp

21. engage*.mp

22. or/20-21

23. 19 and 22

17. 12 and 23
