# Supplementary material for: Interventions to promote patients and families’ involvement in adult intensive care settings: a protocol for a mixed-method systematic review
Source: Syst Rev. 2019 Jul 25;8:185. doi: 10.1186/s13643-019-1102-9 (PMC6657078; doi:10.1186/s13643-019-1102-9)
Supplement: Supplementary file 3 — Draft—data extraction form. (DOCX 17 kb) [file 13643_2019_1102_MOESM3_ESM.docx]

**Additional file 3: Draft – Data extraction form**

| CRITERIA | COMMENTS |  |
| --- | --- | --- |
| Ref. No: |  |  |
| Citation | Author(s). Year. Title. Publication |  |
| Type (jnl, grey lit) | State whether paper has been obtained from a journal/grey literature |  |
| ***Intervention*** | | |
| Aim/objective | | State aims/objectives of intervention |
| Content | | Main component |
| Duration | | Months, Days, hours … |
| Activities | | Specific activities involved in intervention |
| Setting | | ICU type, e.g. surgical, cardiothoracic, generic, open/closed-type  Also country of origin (US, UK, etc) |
| Participants | | State number patients, family members and professionals |
| Context | | Add any contextual information on related to development, implementation or evaluation of intervention |
| ***Method*** | |  |
| Approach to evaluation | | Research design, sampling, data collection method(s), analysis  Also complete quality appraisal form |
| **Outcomes** | | |
| Range/Type | | List all reported outcomes (e.g. SF 36, Impact of Event Scale, Hospital Anxiety and Depression Scale) associated with intervention.  Also note if outcome is self-reported or observed |
