# Supplementary material for: Interventions to promote patients and families’ involvement in adult intensive care settings: a protocol for a mixed-method systematic review
Source: Syst Rev. 2019 Jul 25;8:185. doi: 10.1186/s13643-019-1102-9 (PMC6657078; doi:10.1186/s13643-019-1102-9)
Supplement: Supplementary file 4 — Draft—quantitative quality assessment form. (DOCX 19 kb) [file 13643_2019_1102_MOESM4_ESM.docx]

**Additional file 4: Draft – Quantitative quality assessment form**

| CRITERIA | COMMENTS |
| --- | --- |
| Ref. No: |  |
| Citation | Author(s). Year. Title. Publication |
| Type (jnl, grey lit) | State whether paper has been obtained from a journal/grey literature |
| **Quantitative study designs** | |
| Aim of Evaluation (Implicit/explicit) | What are the evaluation aim(s)?  (Also state whether aims of evaluation implicitly or explicitly stated) |
| Research Design | RCT, CCT, CBA, ITS; other –longitudinal, contemporaneous, retrospective – specify when, only post-PFMI |
| Data collection method | SPECIFIC TYPES -Scales, questionnaires, other measures – structured observation, patient records |
| Source of data | Whether from professionals, patients, family members, author’s description |
| Data analysis method | Descriptive, inferential – detail of type |
| Ethics | Have the relevant ethical issues been discussed (e.g. ethical approval obtained)? Provide details |
| Relevance | In terms of contributing new insights? Suggesting further research? impacting on policy/practice? |
| Number of groups  (in study) | How many arms in study - e.g. 1 intervention, 2 controls |
| Unit of study | (1,2 or more levels ) i.e. individuals as patients or professionals and/or clusters - e.g. ICUs, hospitals, groups of patients or professionals |
| Method of allocation | Describe how subjects allocated to group. |
| Allocation concealment | Judge if the study was designed in such a way that the researchers could not introduce bias which subjects went into the intervention group, or the other group  Score adequate (A), unclear (B), inadequate (C) or not used (D). |
| Blinding | Judge if subjects and evaluators unaware of which subjects got intervention  Score adequate (A), unclear (B), inadequate (C) or not used (D). |
| Power calculation | Was there a calculation made of how big the study needed to be to detect the expected difference? |
| (Original) Sample size | How many participants in each group? |
| Loss to follow up | How many dropouts from each group |
| Significance measures | The mean and the standard deviation (SD), confidence interval for the mean difference, test statistic (t test, F test, chi-square test, etc.) or a p-value |
| Reported biases or confounders | Include bias or confounders reported or identified |
| Strength of design | Although problematic to score the strength of a research of a score between 1-4, this categorisation system should allow a rough idea of research design strength. Score as follows:  • RCT, CBS, ITS =4  • Longitudinal, BA, BDA with control group =3  • BA, BDA =2  • Post intervention study =1 |
| Strength of no. | A score (from 1-3) of the strength of numbers of patients/ family members/ professions involved in study; numbers below informed by previous scoping review. Score as follows:   - more than 30 participants from at least two groups (professionals, patients, family members/carers) =3 - between 10-30 participants from at least two groups =2 - below 10 participants from at least two groups =1 |
| Quality of study | Degree of appropriateness of design in relation to aims of evaluation; clear selection criteria; sources of bias/extraneous factors.  On a scale 5 to 1 (lowest =1) - based on judgement. |
| Quality of information | Clarity of evaluative procedure including internal validity and reliability of measures; significance related to response rate/ effect size. Clarity of context descriptives.  On a scale of 5 to 1 (lowest =1) - this is again judgmental. |
| Overall weighting | This figure is calculated by adding together above four scores (i.e. strength of design, strength of numbers, quality of study, and quality of information) to provide an overall weighting for each paper. |
