# Supplementary material for: Interventions to promote patients and families’ involvement in adult intensive care settings: a protocol for a mixed-method systematic review
Source: Syst Rev. 2019 Jul 25;8:185. doi: 10.1186/s13643-019-1102-9 (PMC6657078; doi:10.1186/s13643-019-1102-9)
Supplement: Supplementary file 5 — Draft—qualitative quality assessment form. (DOCX 16 kb) [file 13643_2019_1102_MOESM5_ESM.docx]

**Additional file 5: Draft – Qualitative quality assessment form**

| CRITERIA | COMMENTS |  |  |
| --- | --- | --- | --- |
| Ref. No: |  |  |  |
| Citation | Author(s). Year. Title. Publication |  |  |
| Type (jnl, grey lit) | State whether paper has been obtained from a journal/grey literature |  |  |
| **Qualitative study designs** | | |  |
| Aim of Evaluation | What are the evaluation aim(s)? Are aims implicitly or explicitly stated? | | |
| Sampling | How were the participants identified and recruited? -Number? Justification? Saturation of data? Appropriate to address research aim(s)? | | |
| Data collection | Observations, semi-structured interviews, focus groups, etc? Research setting/context? How data recorded? Data collection modified during research? Data triangulation? Data collection address research aim(s)? | | |
| Data analysis | How was data analysed? (e.g. thematic, framework, content). Adequate description? Creditability tests? Analysed with sufficient rigor? If so, how? | | |
| Research relations | Researcher considered their own role with participants? Considered own biases? If so how? Relationship with research setting? | | |
| Ethics | Have the relevant ethical issues been discussed (e.g. ethical approval obtained)? Provide details | | |
| Findings | Clearly presented findings? Sufficient data/quotes provided to support findings? What criteria given for selecting examples from data? | | |
| Transferability | Sufficient details from research setting/context to determine similarities/differences? How transferable are finding to wider populations/settings? | | |
| Relevance & Usefulness | In terms of contributing new insights? Suggesting further research? impacting on policy/practice? | | |
| Quality of study | Based on the study design, data collection etc. Assess appropriateness of research design to study aims, issues of typicality, issues of reflexivity, ethical considerations and relevance to different stakeholders. On a scale 5 to 1 (lowest =1) | | |
| Quality of information | For example, clarity of approach, sampling, analysis, significance i.e. impact of study. Clarity of context descriptives. On a scale of 5 to 1 (lowest =1) | | |
| Overall weighting | Combined the two scores together (quality of study and quality of information) to provide an overall weighting for each paper. | | |
